# Expression patterns of protein kinase D 3 during mouse development

**DOI:** 10.1186/1471-213X-8-47

**Published:** 2008-04-25

**Authors:** Kornelia Ellwanger, Klaus Pfizenmaier, Sylke Lutz, Angelika Hausser

**Affiliations:** 1 Institute of Cell Biology and Immunology, University of Stuttgart, Allmandring 31, 70569 Stuttgart, Germany

## Abstract

**Background:**

The PKD family of serine/threonine kinases comprises a single member in *Drosophila *(dPKD), two isoforms in *C. elegans *(DKF-1 and 2) and three members, PKD1, PKD2 and PKD3 in mammals. PKD1 and PKD2 have been the focus of most studies up to date, which implicate these enzymes in very diverse cellular functions, including Golgi organization and plasma membrane directed transport, immune responses, apoptosis and cell proliferation. Concerning PKD3, a role in the formation of vesicular transport carriers at the trans-Golgi network (TGN) and in basal glucose transport has been inferred from *in vitro *studies. So far, however, the physiological functions of the kinase during development remain unknown.

**Results:**

We have examined the expression pattern of PKD3 during the development of mouse embryos by immunohistochemistry. Using a PKD3 specific antibody we demonstrate that the kinase is differentially expressed during organogenesis. In the developing heart a strong PKD3 expression is constantly detected from E10 to E16.5. From E12.5 on PKD3 is increasingly expressed in neuronal as well as in the supporting connective tissue and in skeletal muscles.

**Conclusion:**

The data presented support an important role for PKD3 during development of these tissues.

## Background

The protein kinase D (PKD) family of serine/threonine kinases comprises a single member in *Drosophila *[1,2], two isoforms in *C. elegans *[3,4] and three members, PKD1 (PKCμ), PKD2 and PKD3 (PKCν) in mammals. The three mammalian isoforms share similar structural modules. They consist of an N-terminal regulatory domain and a C-terminal kinase domain. While PKD1 and PKD2 posses an alanine/proline-rich region at their N-terminus, in PKD3 this hydrophobic domain is absent. All isoforms contain two cysteine-rich domains (CRD) separated by a long linker region, an acidic region consisting of negatively charged amino acids and a pleckstrin homology domain (PH). These characteristic motifs are also important for the regulation of enzyme activity and localization within cells. The PKD enzymes have recently been implicated in very diverse cellular functions, including Golgi organization and plasma membrane directed transport, metastasis, immune responses, apoptosis and cell proliferation (for an overview see [5]). PKD3 was originally identified in 1999 [6]. Northern blot analysis revealed a ubiquitous expression of the protein in a wide variety of human tissues suggesting a basic housekeeping function [6]. *In vitro *studies propose a potential role of the kinase in signaling events of GPCR agonists which induced a rapid activation of PKD3 by a protein kinase C (PKC)-dependent pathway [7]. PKD3 can also be activated by bombesin in a Rac and Gα dependent mechanism [8,9]. Moreover, PKD3 was implicated to play a role in B cell antigen receptor signaling by phosphorylating class II HDAC5 and 7 thereby promoting nuclear export of these proteins [10,11]. Activation of PKD3 in these cells involves the phosphorylation of the activation loop serines which is mediated by a DAG-PLC-PKC-dependent pathway. Putative upstream kinases for PKD3 are PKCε, PKCη or PKCθ [10]. According to the localization of the kinase at the trans-Golgi network (TGN) [12], a function in the formation of exocytotic transport carriers has been described [13]. Recently, it could be demonstrated that PKD3 and PKD2 dimerize at the TGN and regulate membrane fission [14]. However, there is also substantial evidence that PKD3 has a distinct and non-redundant function within the PKD family. Compared to PKD1 and 2, PKD3-specific direct substrates at the TGN have not been identified yet [12,15]. A PDZ domain identified in PKD1 and 2 is lacking in PKD3 [16]. Moreover, PKD1 and PKD2, but not PKD3, are targets for PKCδ in response to oxidative stress, because PKD3 lacks the relevant tyrosine residue that generates a PKCδ interaction motif [17]. PKD3 also localizes to vesicular structures that are part of the endocytic compartment. This localization may be mediated by the interaction of PKD3 with the vesicle-associated membrane protein VAMP2 [18]. In L6 skeletal muscle cells, a specific role for PKD3 in basal glucose uptake could be demonstrated [19].

The functional activities of PKD3 described so far are derived from *in vitro *studies performed with established cell lines. Transgenic mouse models, which allow interference with endogenous PKD3, are not available. Consequently, the *in vivo *functions of PKD3 remain unknown so far. In a recent report, Oster and colleagues described the expression of PKD isoforms during mouse embryogenesis using *in situ *hybridization techniques [20]. In early stages of development, PKD3 mRNA was clearly detected in the heart, nasal processes and limb buds. During later stages of development, PKD3 transcript was more or less ubiquitously present. In an independent study, we have investigated the expression of PKD3 protein by immunohistochemistry on sagittal sections of mouse embryos using a PKD3 specific antibody. We here describe PKD3 protein expression in developmental stage E10 through E16.5 of the mouse.

## Results and Discussion

### Specificity of the PKD3 antibody

**Figure 1:**
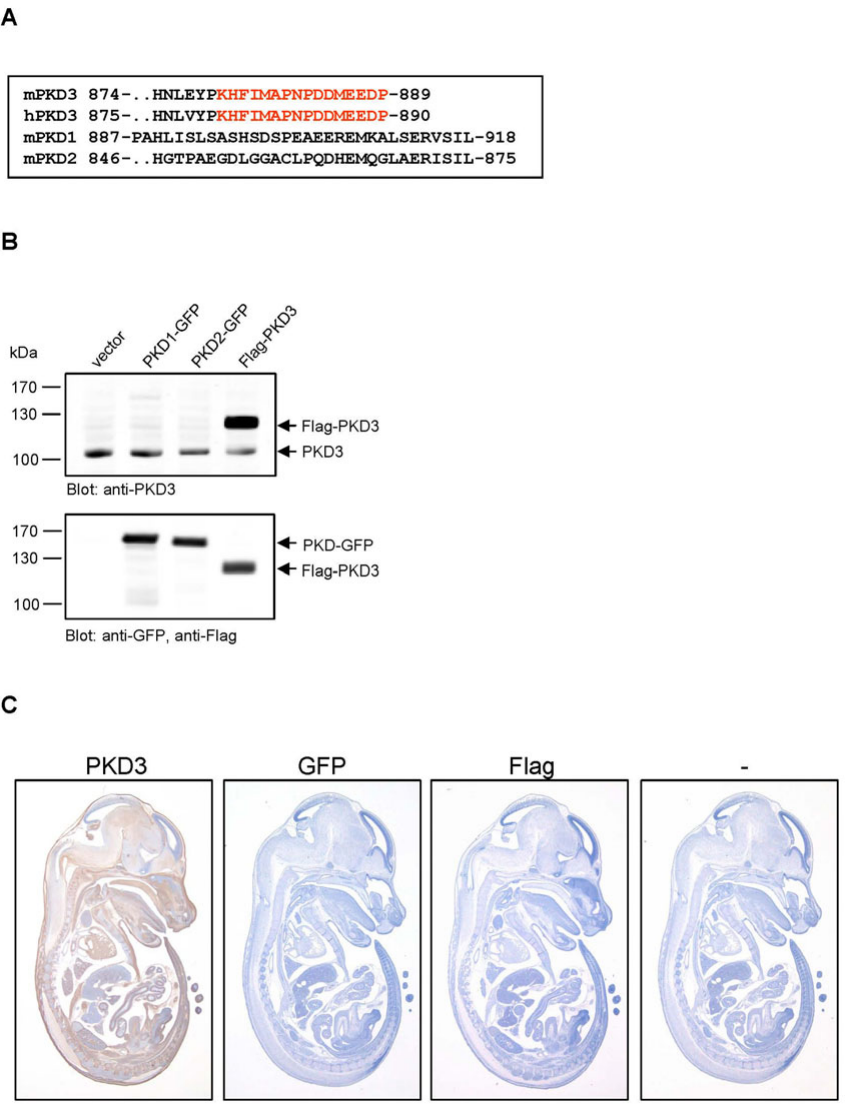
**Specificity of the PKD3 antibody**. **A**: Sequence alignment of mouse PKD3 with human PKD3, mouse PKD1 and PKD2. The sequence of PKD3 used for peptide synthesis and immunisation of mice is marked in red. **B**: HEK293T cells were transfected with the indicated plasmids, whole cell lysates were prepared and Western blot analysis was performed with the indicated antibodies. **C: **Immunohistochemistry on sagittal sections (6 μm) of E14.5 mouse embryos. Sections incubated with mouse Flag- and rabbit GFP-specific antibodies (both 0.5 μg/ml), respectively or without primary antibody (-) were negative and used as control. Section incubated with rabbit anti-PKD3 antibody (1:2000) displayed specific staining.

The affinity purified polyclonal PKD3 specific antibody used in this study was generated by immunization of rabbits with a peptide corresponding to the C-terminal epitope of human PKD3 (amino acids 875 to 890). This epitope is conserved in the mouse orthologous gene, but not present in PKD1 or PKD2 isoforms (Fig. 1A). To demonstrate that the antibody is specific for PKD3 and not cross-reactive with PKD1 and PKD2, we performed Western blot analysis. Total cell lysates of HEK293T cells transiently transfected with plasmids encoding GFP-tagged human PKD1, GFP-tagged human PKD2, and human Flag-tagged PKD3 were analyzed (Fig. 1B). The PKD3-specific antibody detected Flag-PKD3, migrating at about 120 kDa, and endogenous PKD3, migrating at about 105 kDa, but not PKD1 or PKD2, thus demonstrating the specificity of the reagent. Since no PKD3 mutants are available, the antibody could not be tested in a PKD3 deficient background. We have therefore applied several approaches to confirm specificity of the PKD3 antibody used here; control staining procedures performed without the primary antibody or with rabbit preimmune-serum were all negative. Various antibody dilutions (1:500 – 1:2000) had no effect on the staining pattern (data not shown). In addition, we performed staining with either Flag-specific mouse monoclonal or GFP-specific rabbit polyclonal antibodies that was negative, too (Fig. 1C).

### PKD3 expression in early and later stages of development

Performing immunohistochemistry on paraffin sections of mouse embryos at developmental stage E10 – E16.5 we obtained histological details of PKD3 expression.

**Figure 2:**
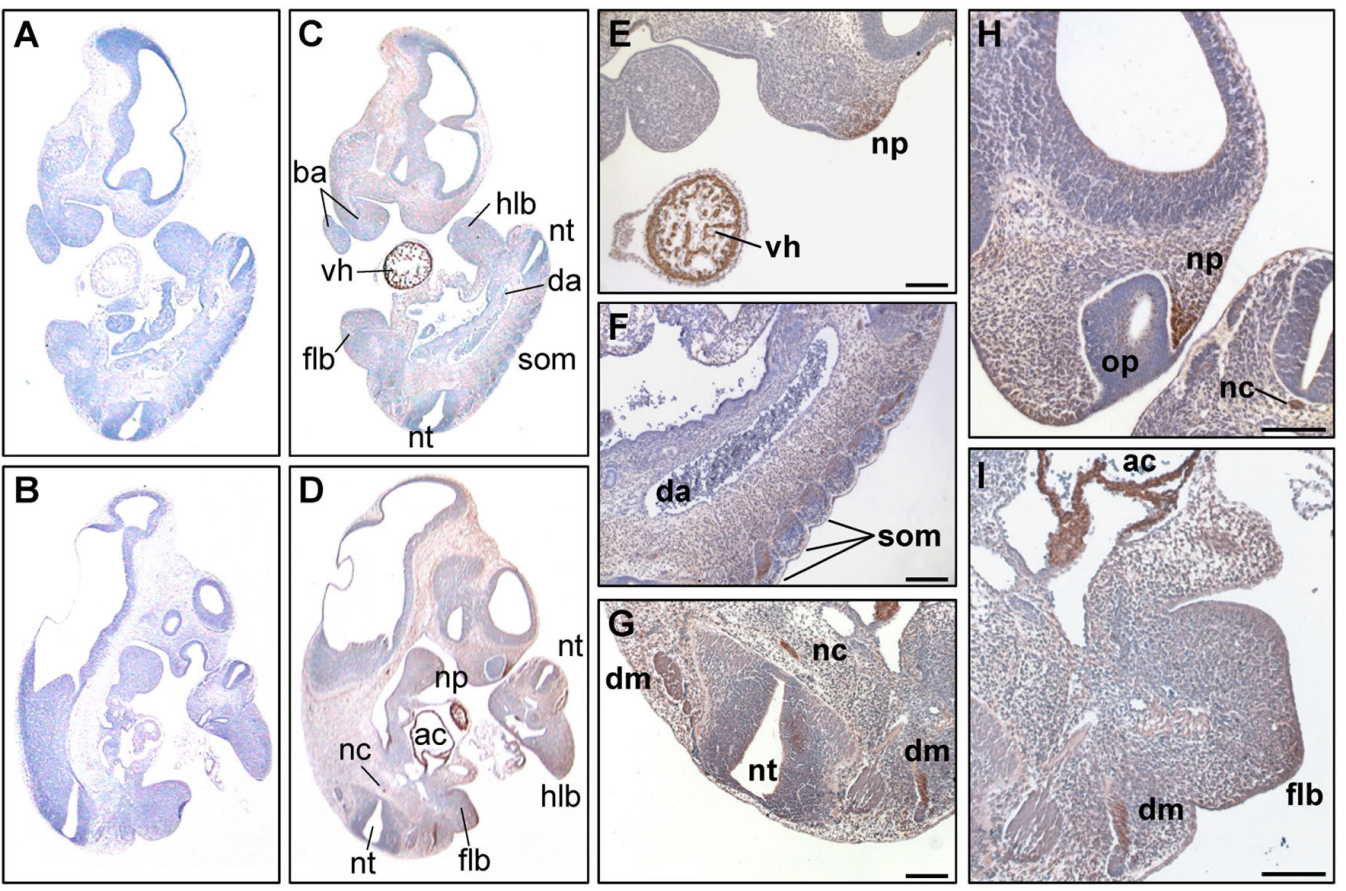
**PKD3 expression at embryonic stage E10**. Immunohistochemistry was performed on sagittal sections (6 μm) of E10 mouse embryos. (A) – (B) Control sections incubated without primary antibody. (C) – (I) Sections incubated with anti-PKD3 antibody (1:2000). ac: atrial chamber, ba: branchial arch, da: dorsal aorta, dm: dermomyotome, flb: forelimb bud, hlb: hindlimb bud, nc: notochord, np: nasal process, nt: neural tube, op: olfactory pit, som: somites, vh: ventricular chamber of heart. Scale bars: 100 μm.

**Figure 3:**
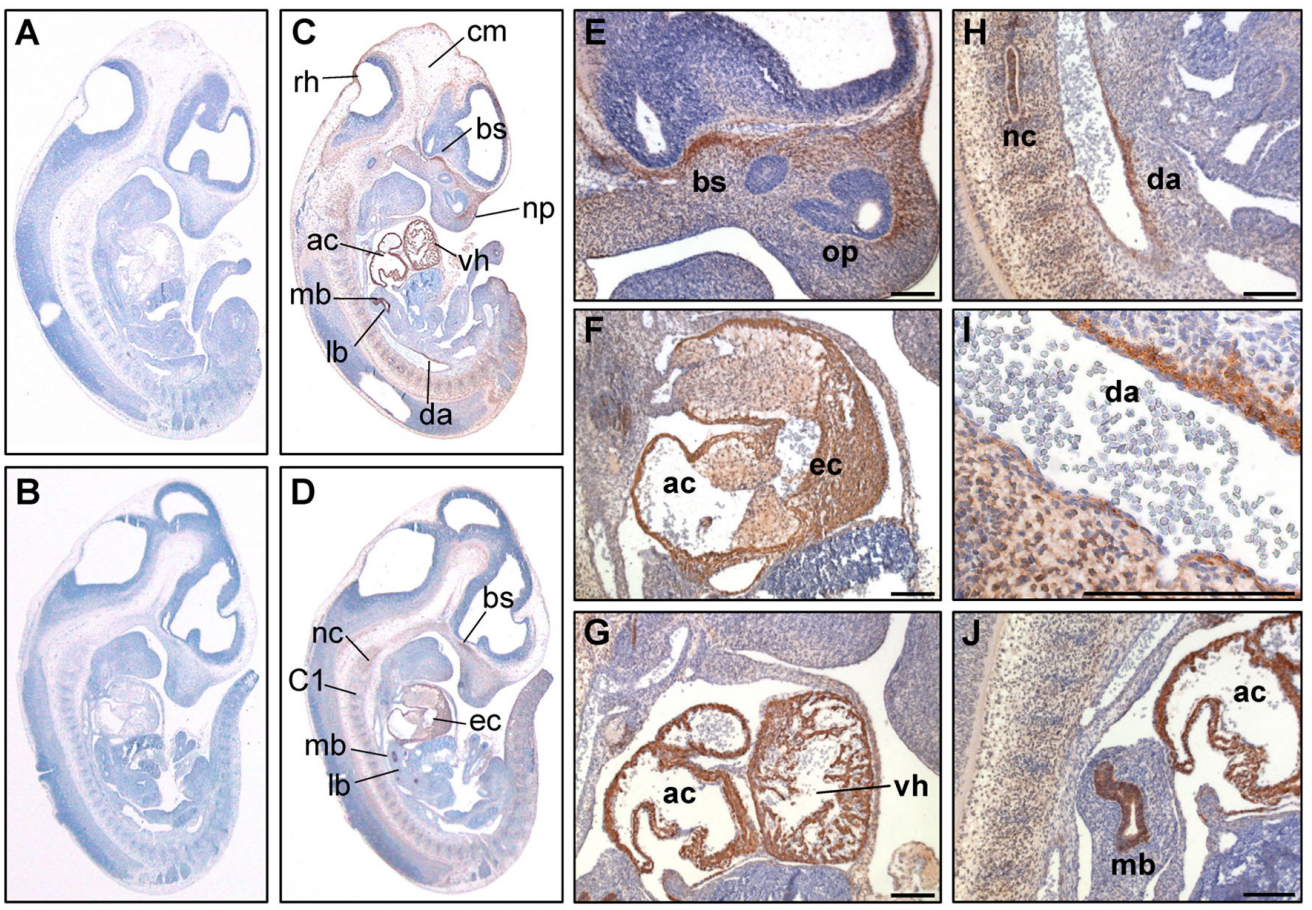
**PKD3 expression at embryonic stage E11.5**. Immunohistochemistry was performed on sagittal sections (6 μm) of E11.5 mouse embryos. (A) – (B) Control sections incubated without primary antibody. (C) – (J) Sections incubated with anti-PKD3 antibody (1:2000). ac: atrial chamber, bs: cartilage primordium of base of the skull, C1: condensation of sclerotomic material forming centrum of atlas, cm: cephalic mesenchyme, da: dorsal aorta, ec: endocardial cushion tissue lining the atrio-ventricular canal, lb: lung bud, mb: main bronchus, nc: notochord, np: nasal process, op: olfactory pit, rh: roof of hindbrain, vh: ventricular chamber of heart. Scale bars: 100 μm.

**Figure 4:**
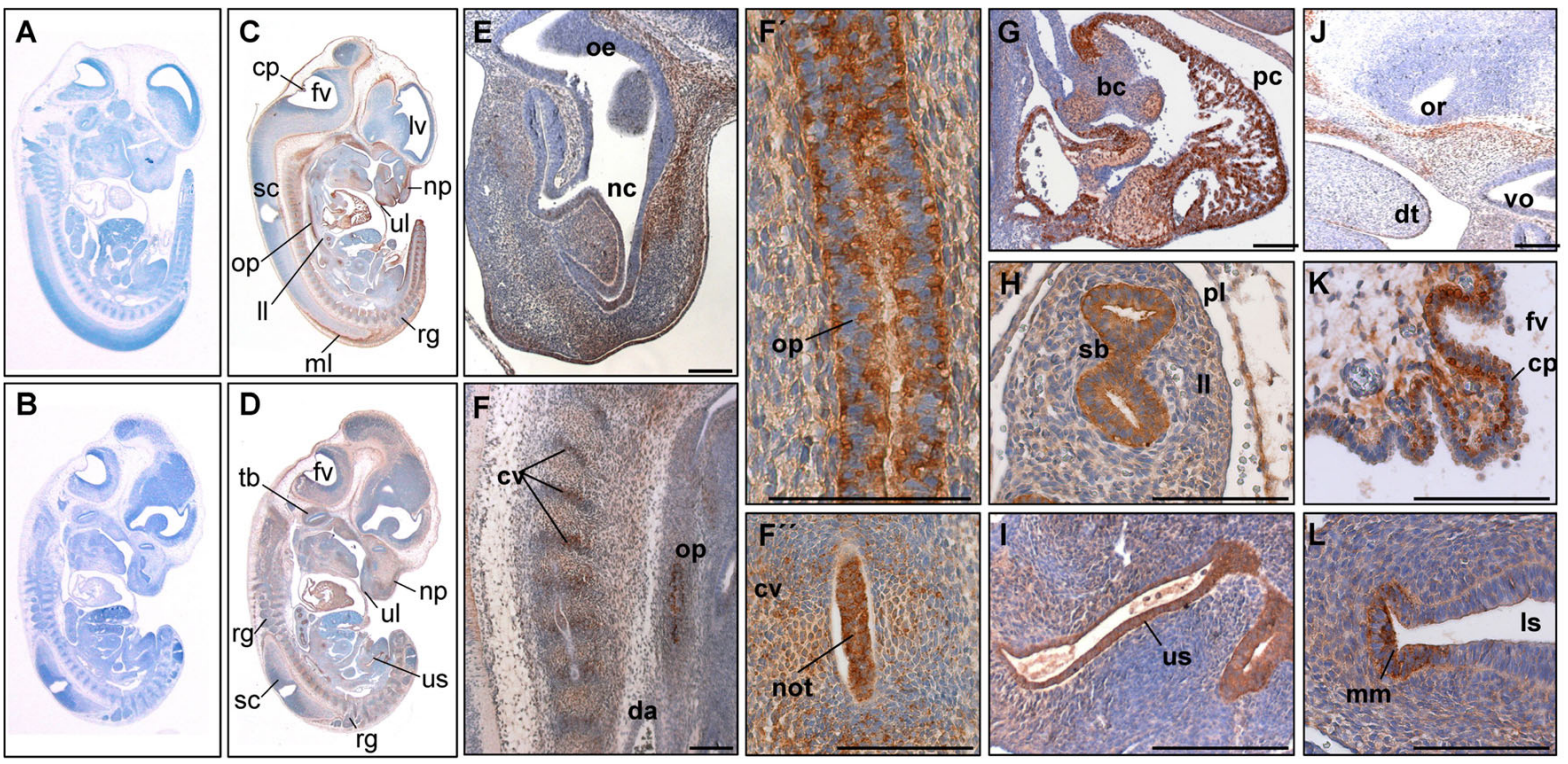
**PKD3 expression at embryonic stage E12.5**. Immunohistochemistry was performed on sagittal sections (6 μm) of E12.5 mouse embryos. (A) – (B) Control sections incubated without primary antibody. (C) – (L) Sections incubated with anti-PKD3 antibody (1:2000). bc: atrio-ventricular bulbar cushion tissue, cp: origin of choroid plexus, cv: cartilage primordium of vertebra, da: descending (thoracic) aorta, dt: dorsum of tongue, fv: fourth ventricle, ll: left lung, ls: lumen of stomach, lv: lateral ventricle, ml: marginal layer of spinal cord, mm: mucous membrane, nc: nasal cavity, not: notochord, np: nasopharynx, oe: olfactory epithelium, op: oesophagus, or: optic recess of diencephalon, pc: pericardial cavity, pl: pleural cavity, rg: root ganglia, sb: segmental bronchus, sc: spinalcord, tb: cartilage primordium of temporal bone, ul: upper lip, us: urogenital sinus, vo: vomeronasal organ. Scale bars: 100 μm.

**Figure 5:**
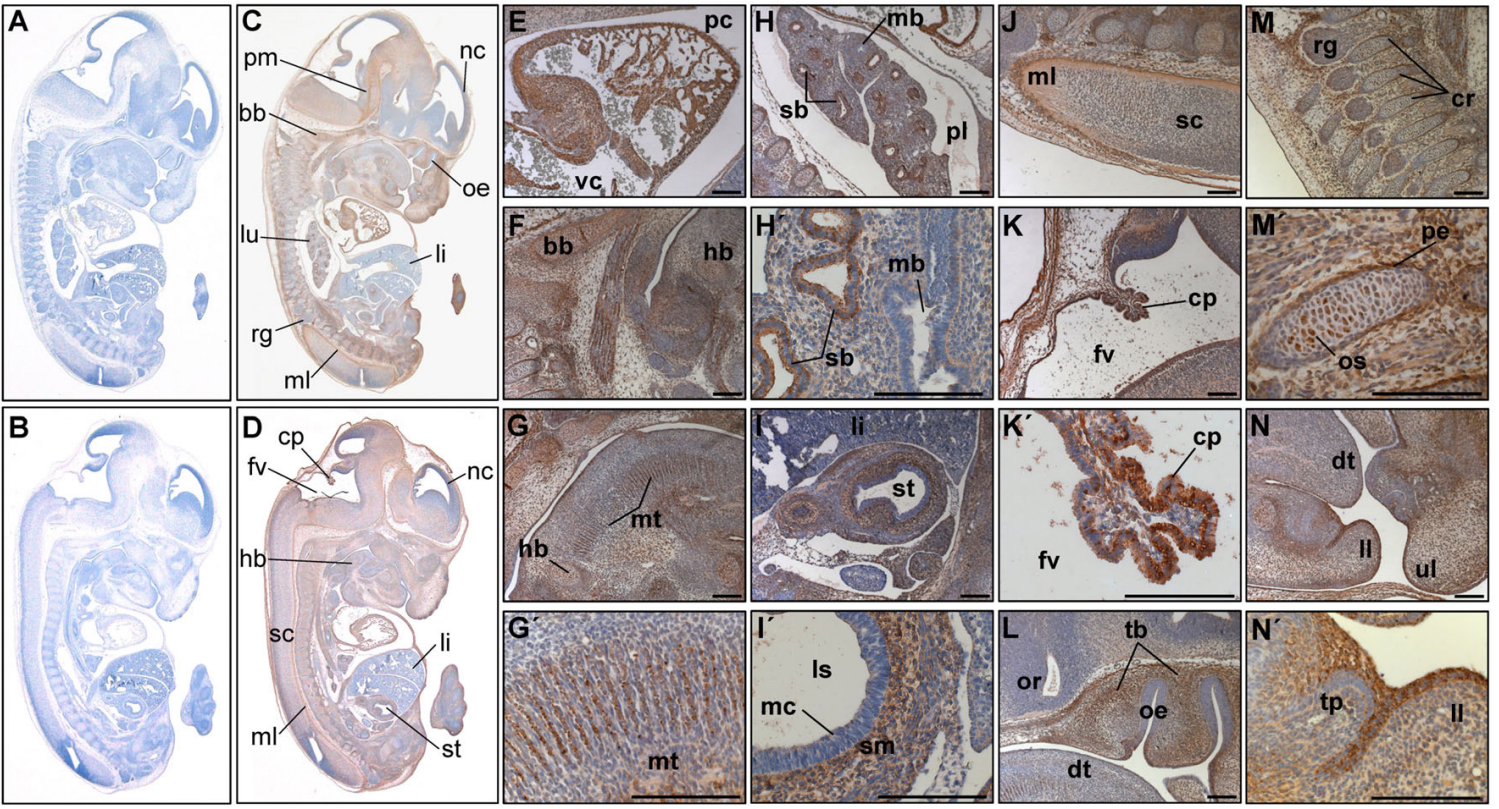
**PKD3 expression at embryonic stage E13.5**. Immunohistochemistry was performed on sagittal sections (6 μm) of E13.5 mouse embryos. (A) – (B) Control sections incubated without primary antibody. (C) – (N') Sections incubated with anti-PKD3 antibody (1:2000). bb: cartilage primordium of basioccipital bone (clivus), cp:choroid plexus, cr: cartilage primordium of ribs, dt: dorsum of tongue, fv: fourth ventricle, hb: cartilage primordium of body of hyoid bone, li: liver, ll: lower lip, ls: lumen of stomach, lu: lung, mb: main bronchus, mc: mucosal lining, ml: marginal layer of spinal cord, mt: muscle mass of the tongue, nc: neocortex, oe: olfactory epithelium, or: optic recess of diencephalon, os: osteoblasts, pc: pericardial cavity, pe: perichondrium, pl: pleural cavity, pm: pons-midbrain junction, rg: root ganglion, sb: segmental bronchus, sc: spinal cord, sm: submucosa, st: stomach, tb: cartilage primordium of turbinate bones, tp: tooth primordium, ul: upper lip, vc: vena cava. Scale bars: 100 μm.

**Figure 6:**
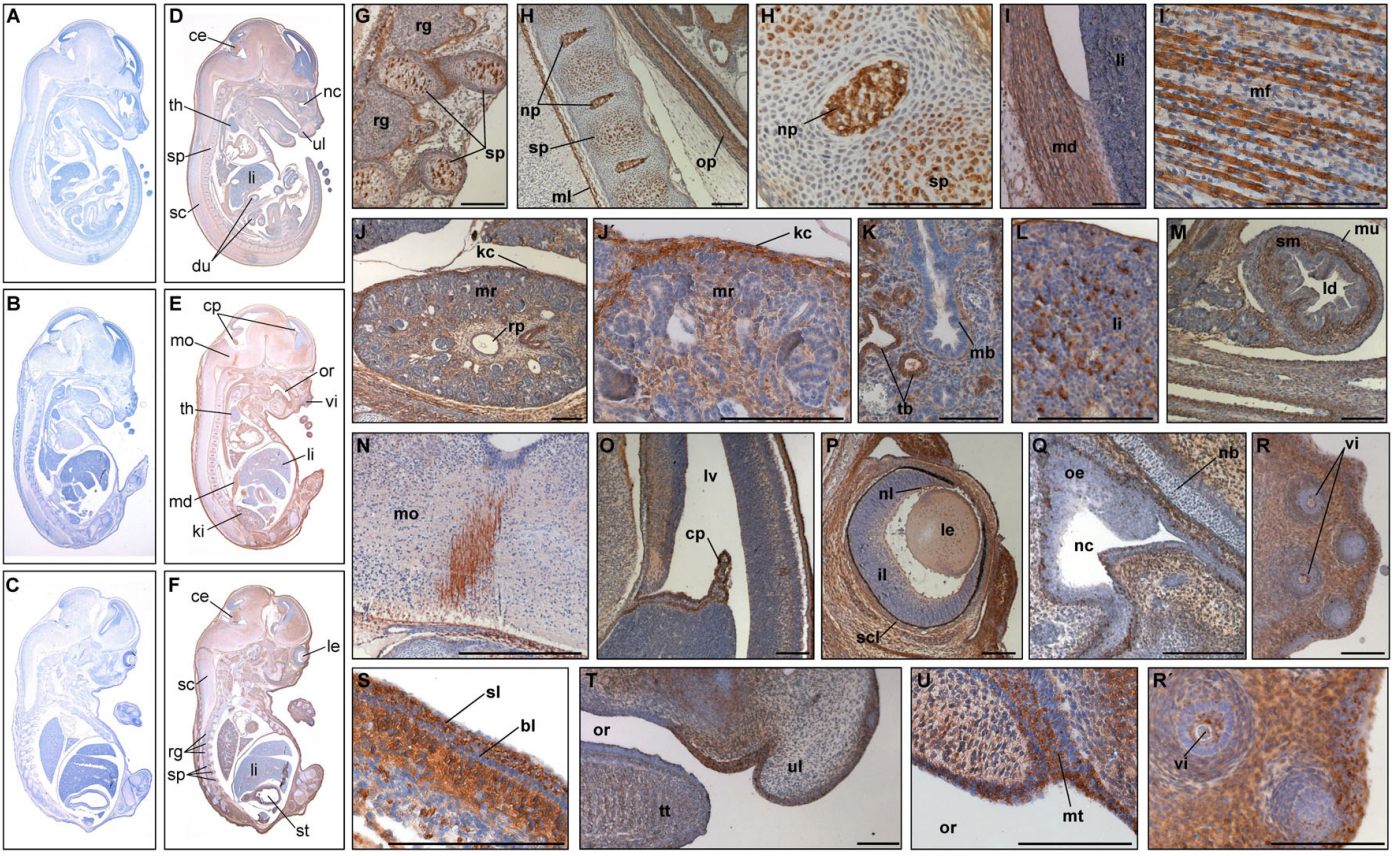
**PKD3 expression at embryonic stage E14.5**. Immunohistochemistry was performed on sagittal sections (6 μm) of E14.5 mouse embryos. (A) – (C) Control sections incubated without primary antibody. (D) – (U) Sections incubated with anti-PKD3 antibody (1:2000). bl: basal layer of epidermis, ce: cerebellum, cp: choroid plexus, du: duodenum, il: inner layer of retina, kc: kidney capsule, ki: kidney, ld: lumen of duodenum, le: lens, li: liver, lv: lateral ventricle, mb: main bronchus, md: muscle of diaphragm, mf: muscle fibers, ml: marginal layer of spinal cord, mo: medulla oblongata, mr: medulla renalis, mt: primordium of upper molar tooth, mu: muscularis layer of duodenum, nb: cartilage primordium of the nasal bone, nc: nasal cavity, nl: neural layer of retina, np: nucleus pulposus in the central region of future invertebral disc, oe: olfactory epithelium, op: oesophagus, or: oropharynx, rg: root ganglia, rp: renal pelvis, sc: spinal cord, scl: sclera, sl: suprabasal layer of epidermis, sm: submucosal layer of duodenum, sp: cartilage primordium of spinal column, st: stomach, tb: terminal bronchus, th: thymus, tt: tip of the tongue, ul: upper lip, vi: primordia of follicles of vibrissae. Scale bars: 100 μm.

**Figure 7:**
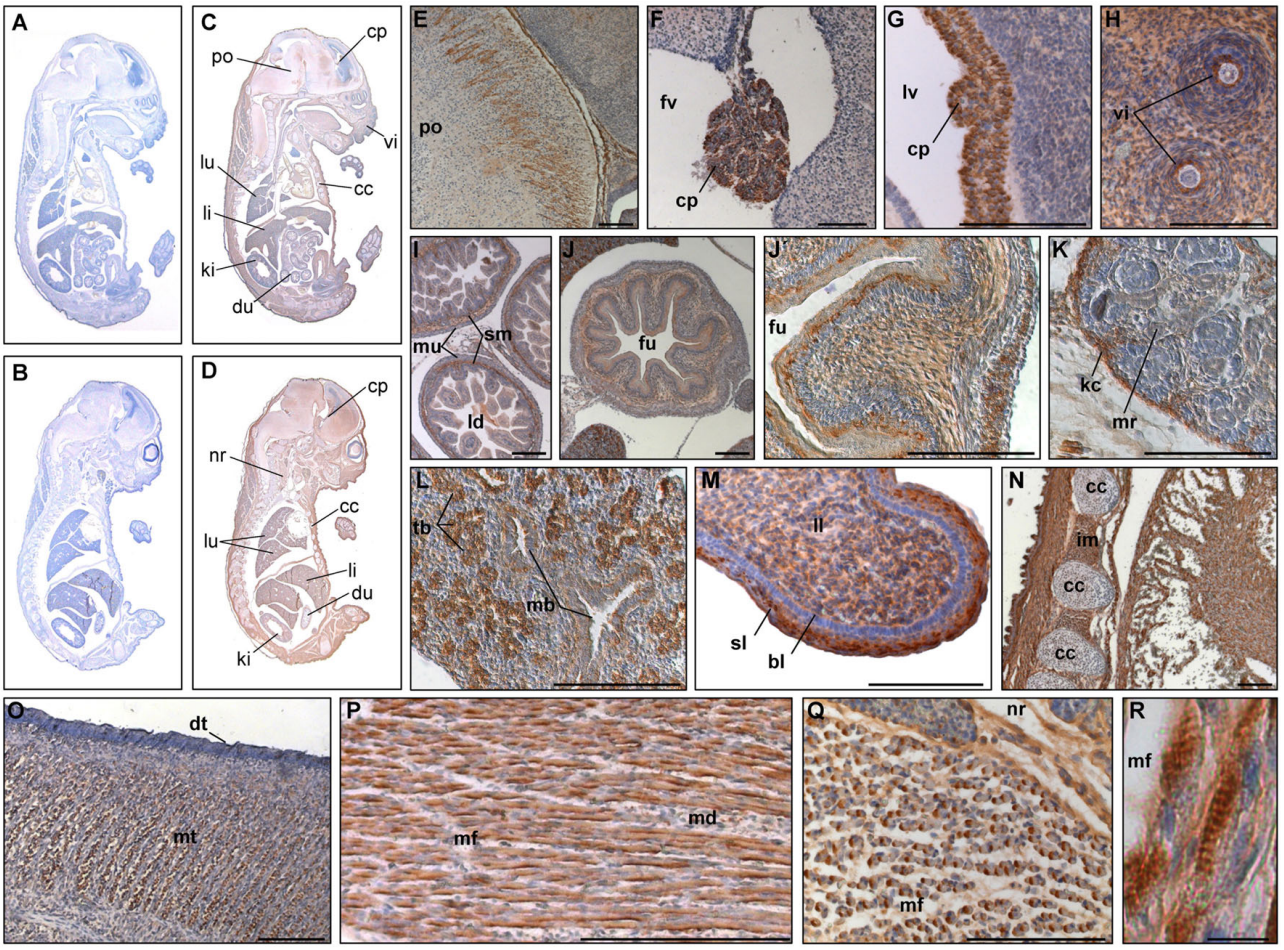
**PKD3 expression at embryonic stage E16.5**. Immunohistochemistry was performed on sagittal sections (6 μm) of E16.5 mouse embryos. (A) – (B) Control sections incubated without primary antibody. (C) – (Q) Sections incubated with anti-PKD3 antibody (1:2000). bl: basal layer of epidermis, cc: costal cartilage, cp: choroid plexus, dt: dorsum of tongue, du: duodenum, fu: fundus region of stomach, fv: fourth ventricle, im: intercostal muscle, kc: kidney capsule, ki: kidney, ld: lumen of duodenum, li: liver, ll: lower lip, lu: lung, lv: lateral ventricle, mb: main bronchus, md: muscle fibers of diaphragm, mf: muscle fibers, mr: medulla renalis, mt: muscle mass of the tongue, mu: muscularis layer, nr: neck region, po: pons, sl: suprabasal layer of epidermis, sm: submucosal layer, tb: terminal bronchus, vi: primordia of follicles of vibrissae. Scale bars: 100 μm (E) – (Q), 10 μm (R).

In E10.0 the most prominent expression was evident in the developing heart (Fig 2C, D and 2E). Cells forming the ventricular and atrial chambers showed a strong staining (Fig. 2E and 2I). This strong expression in the heart was visible from early to late stages of embryonic development (Fig. 2, 3, 4, 5, 6, 7). Recent work suggests an important role for the PKD kinase family in cardiac myocytes. The expression of PKD1 and PKD2 or PKD3 was demonstrated in neonatal and adult rat ventricular myocytes as well as adult mouse, rat, rabbit, and human myocardium (for an overview see [21]). Of note, the PKD expression seems to be subject to developmental regulation and declines significantly in adulthood [22]. Furthermore, studies with transgenic mice revealed a role for PKD1 in pathological cardiac remodeling. Cardiac-specific expression of a constitutive active PKD1 *in vivo *caused hypertrophy, chamber dilation, and impaired systolic function [23]. Conversely, mice with a cardiac specific PKD1 knock-out demonstrated an impaired response to stress signals that normally lead to cardiac hypertrophy, fibrosis and fetal gene activation [24]. There is substantial evidence that this phenotype is associated with the PKD-mediated phosphorylation of class II HDAC5. However, each PKD isoform is capable of phosphorylating class II HDACs on the serines that mediate nuclear export via interaction with 14-3-3 proteins [11,25], suggesting that PKD family members could act redundant. Although the diminished hypertrophic response of PKD1 cardiac knock-out mice indicates that PKD2 and 3 cannot fully compensate for the loss of PKD1 it is likely that the residual hypertrophy and fetal gene activation in these animals reflects redundant functions of cardiac PKD2 and 3.

In addition, PKD3 was expressed in the nasal processes (Fig. 2D, E and 2H) and forelimb buds (Fig. 2I). These observations are in line with previously published data on mRNA distribution at this stage [20]. On top of this, an obvious PKD3 expression was also visible in the embryonic mesoderm. Especially somite derived structures forming the dermomyotome (Fig. 2F, G and 2I) and the notochord (Fig. 2G, H) were PKD3 positive. Of note, PKD3 was not expressed in erythrocytes present in the atrium and the dorsal aorta (Fig. 2F and 2I). In contrast to *in situ *hybridization studies [20] PKD3 protein could not be detected in the forebrain or midbrain region at this stage. This might be due to low levels of PKD3 mRNA at this stage [20], which might result in low protein levels that are difficult to detect and/or additional regulation of PKD3 expression at the posttranscriptional level in this tissue.

In embryonic stage E11.5 PKD3 expression was detectable in additional tissues (Fig. 3C and 3D). Parts of the head region near the developing base of the scull and nasal process (Fig. 3E) were positive for PKD3 protein. PKD3 expression was visible in and along the notochord, where sclerotomic material is condensed to form the centrum of the axis (Fig. 3H and 3J). A strong PKD3 expression was detected in the bronchus of the lung bud (Fig. 3J) restricted to the cytoplasm of the epithelium. PKD3 expression in the cardiac muscle cells was still obvious (Fig. 3F, G). Moreover, PKD3 was expressed in the wall of the dorsal aorta at this stage (Fig. 3H and 3I).

In embryonic stage E12.5 more cytological details of PKD3 expression were visible (Fig. 4C, D). PKD3 was detected in the cartilage primordium of nasal bones (Fig. 4E), the temporal bones (Fig. 4J) and the vertebra (Fig. 4F). PKD3 expression was still detectable in the notochord (Fig. 4F and 4F") and in cardiomyocytes forming the ventricular and atrial chambers but was absent from the atrio-ventricular bulbar cushion tissue (Fig. 4G). Further, the membrane of the oesophagus showed a strong PKD3 expression (Fig. 4F and 4F'). Within the lung strong PKD3 expression was found in epithelial cells of segmental bronchii (Fig. 4H). PKD3 expression is also observed in the urogenital ridge surrounding the lumen of the urogenital sinus (Fig. 4I). Moreover, PKD3 expression now became detectable in the developing brain, with a prominent staining of the outer layer of the choroid plexus within the fourth ventricle (Fig. 4K). In the stomach, the mucous membrane was PKD3 positive (Fig. 4L).

In embryonic stage E13.5 PKD3 was found to be more or less ubiquitously expressed (Fig. 5C and 5D). In addition to PKD3 positive structures like cardiomyocytes (Fig. 5E), the protein could also be detected in further muscle structures: Skeletal muscle cells of the neck region (Fig. 5F) as well as muscles of the tongue (Fig. 5G and 5G') showed strong PKD3 expression. In the lung, the epithelial layer of the segmental but not the main bronchi was positive (Fig. 5H, H'). A prominent PKD3 expression could be detected in the middle layer (submucosa) of the stomach, but not the inner layer (mucosa) (Fig. 5I and 5I'). Prominent sites of PKD3 expression were also detected in the marginal layer of the spinal cord (Fig. 5J) and the choroid plexus within the fourth ventricle (Fig 5K and 5K'). Expression of PKD3 was also detected in the developing bones. Especially the cartilage primordium of turbinate bones (Fig. 5L) and osteoblasts in the vertebrae which secrete bone material into previously existing cartilage matrix were intensively stained (Fig. 5M and 5M'). The membranous layer surrounding cartilage during ossification, the perichondrium, was also positive for PKD3 expression (Fig. 5M'). Of note, the ventricular zone of the neocortex as well as the liver was negative (Fig 5C and 5D).

PKD3 expression in osteoblasts was even more prominent in stage E14.5 in developing bones of the spinal column (Fig. 6G). The nucleus pulposus in the middle of the spinal disc was also intensively stained (Fig. 6H, H'). Skeletal muscle cells of the diaphragm (Fig. 6I, I') also showed a strong PKD3 expression. Oster and colleagues failed to detect elevated levels of PKD3 mRNA in skeletal muscle by *in situ *hybridization [20]. The reason for this seeming discrepancy is unclear and might be of technical nature or reflect the fact that the level of mRNA does not necessarily correlate with the protein level.

In this stage we found a strong expression in the kidney capsule (Fig. 6J, J'), in terminal bronchioles of the lung (Fig. 6K), in the nuclei of distinct cells of the liver (Fig. 6L) as well as in the middle layer of the duodenum (Fig. 6M). In the brain, PKD3 expression was ubiquitously detected with exception of the neocortex, which was negative (Fig. 6D, E, F). Interestingly, in the medulla oblongata distinct nerve tracts were intensively stained for PKD3 (Fig. 6N); moreover, the choroid plexus within the fourth and lateral ventricle was also positive (Fig. 6E, O). In accordance with published data on PKD3 mRNA expression [20] the inner layer of the retina shows a weak PKD3 specific staining, whereas the extrinsic ocular muscle displays a strong expression of PKD3 (Fig. 6P). Interestingly, cells within the olfactory epithelium displayed a strong PKD3 signal within the nuclei (Fig. 6Q). The root sheath of the Whisker follicles showed a steady PKD3 expression in E14.5 and E16.5 (Fig. 6R, R', and 7H). Furthermore, the suprabasal layer of the epidermis as well as underlying connective tissue (Fig. 6S) are positive for PKD3. The epidermal staining was impressively evident in the mouth region where PKD3 negative endoderm and PKD3 positive ectoderm derived epidermal layers came into direct contact (Fig. 6T). PKD3 is also expressed in the first primordium of the upper molar tooth (Fig. 6U), which is formed by an incorporation of dental epithelium.

PKD3 was found to be more ore less ubiquitously expressed in embryonic stage E16.5 (Fig. 7C and 7D). Particularly strong PKD3 expression was detected in nerve tracts in the pons (Fig. 7E) and the choroid plexus of the lateral and fourth ventricle (Fig. 7F, G). The middle layer (submucosa) but not the mucosa or the muscle layer (Fig. 7I) demonstrated PKD3 expression. In contrast to E13.5, the submucosa of the stomach showed only a weak PKD3 expression, however, smooth muscle cells in the muscularis layer of the stomach were still PKD3 negative (Fig. 7J, J'). Within the liver PKD3 expression seemed to be further increased (Fig. 7C, D), which is in accordance with high mRNA levels detected by *in situ *hybridization in E18.5 [20]. The strong PKD3 expression in the kidney capsule (Fig. 7K) and in the terminal bronchi of the lung (Fig. 7L) detected in E14.5 was even more evident. In the region of the lower lip, the intensive staining of the suprabasal layer of the epidermis was obvious (Fig. 7M). All skeletal muscle cells within the embryo showed a strong PKD3 expression (Fig 7N–R) e.g. intercostal muscle cells (Fig. 7N) and the transverse muscle fibers of the tongue (Fig. 7O). Interestingly, PKD3 distribution in skeletal muscle cells of the diaphragm (Fig. 7P) and the neck region (Fig. 7Q) is polarized, especially visible in cross-sections (Fig. 7Q). Of note, PKD3 was not detected in smooth muscle cells and in the thymus at this stage. Interestingly, Oster and colleagues could detect PKD3 mRNA in the thymus at E18.5 [20]. In line with this, Western blot analysis revealed a strong PKD3 expression in the thymus of adult animals (unpublished observation K. Ellwanger), suggesting an increase in the expression level of PKD3 protein in neonatal mice.

## Conclusion

The expression pattern of PKD3 reveals a tissue selective expression at stage E10, which became more abundant and distributed later on during embryonic development. Our data are in accordance with previously published results on PKD3 mRNA levels using *in situ *hybridization analysis [20]. On top of that, we provide a comprehensive study on the expression pattern of PKD3 during organogenesis discovering additional histological details. The strong expression of PKD3 in specific tissues, e.g. cardiac and skeletal muscle, points to an important role for this kinase in the development of these tissues. PKD3 has been implicated in the regulation of secretory transport processes at the Golgi compartment [13,14] as well as regulation of basal glucose uptake in skeletal muscle cells [19], both of which are important processes during organogenesis. Moreover, PKD3 has been shown to regulate the nuclear localization and thus activity of its physiological substrates class II HDAC5 and 7 [11]. Interestingly, class II HDAC proteins play an important role in heart development and function [26,27]. It will now be exciting to investigate the potential function of PKD3 in these tissues using transgenic mouse models, interfering with endogenous PKD3 function by overexpression of a dominant-negative protein or deletion of the PKD3 gene (knock-out).

## Methods

### Antibodies

The primary antibody used in this study was an affinity-purified PKD3 specific polyclonal antibody raised in rabbits against a C-terminal epitope of human PKD3 (amino acids 875-890). The mouse monoclonal GFP- and Flag-specific antibodies were obtained from Roche Biosciences and Sigma-Aldrich, respectively. The rabbit polyclonal GFP-specific antibody was from Santa Cruz Biotechnology. The secondary IRdye-conjugated antibodies were from Li-COR Biosciences. Transfection of HEK293T cells was performed as described in [12].

### Animals

C57BL/6 mice were time mated and pregnant females were sacrificed to collect the embryos at different stages (E10 – E16.5). The finding of a vaginal plug at noon was considered as E0. The fetuses were isolated from the uterus and dissected free of embryonic membranes in ice-cold PBS. All animal experiments carried out in this study were approved by the ethical committee at the University of Stuttgart.

### Western Blot

Cells were harvested and lysed in lysis buffer (20 mM Tris (pH7.4), 150 mM NaCl, 1 mM EDTA, 1 mM EGTA, and 1% Triton X-100, plus protease and phosphatase inhibitors). Equal amounts of protein were subjected to a 4–12% NuPAGE Bis-Tris-Gel (Invitrogen, Germany), blotted onto a nitrocellulose membrane and blocked with 0.5% blocking buffer (Roche Biosciences, Germany). Incubation with primary antibodies was performed in blocking buffer at 4°C overnight. After washing with PBS, samples were incubated with secondary IRdye680-conjugated anti-mouse or IRdye800-conjugated anti-rabbit IgG antibodies in blocking buffer for 1 h at room temperature. The detection was performed on an Odyssey Infrared Imaging System (Li-COR Biosciences, Germany).

### Immunohistochemistry

After fixation with paraformaldehyde embryos were dehydrated through a graded ethanol series and into a 50:50 mixture of ethanol:Histoclear (Carl Roth GmbH, Karlsruhe, Germany). Tissue was then incubated in Histoclear for 1–3 hours and transferred into a 50:50 mixture of Histoclear:Paraplast (Carl Roth GmbH, Karlsruhe, Germany) at 42°C, where it was incubated over night. Embryos were transferred into pure Paraplast preheated to 60°C and incubated for 1–3 days. Serial sagittal sections were cut at a thickness of 10 μm on a microtome and floated on a 40°C water bath. Sections were dewaxed in 3 changes of Roticlear for 10 minutes each and incubated in isopropanol and ethanol 15 minutes each. Slides were rehydrated through a reverse series of ethanol dilutions in PBS and finally washed in PBS. Endogenous peroxidase activity was quenched by incubation in PBS containing 0.3% H_2_O_2 _for 30 minutes. Slides were rinsed with PBS for 10 minutes and then incubated with 5% normal goat serum in a humidified chamber to block unspecific binding sites. Primary antiserum diluted in 1.5% normal goat serum was applied. Slides were incubated in the humidified chamber at 4°C over night.

Immunohistochemical staining was performed using the Vectastain Elite ABC Kit (rabbit IgG) (Vector Laboratories, Burlingame, CA, USA). To remove non-specifically bound antibody, slides were washed three times in PBS for 10 minutes each time. Subsequently, sections were incubated with biotinylated goat anti-rabbit IgG diluted 1:200 in 1.5% normal goat serum for 1 hour at room temperature. In case of the mouse monoclonal Flag-specific antibody, a biotin-SP-conjugated goat anti-mouse IgG (Jackson Immunoresearch, West Grove, PA, USA) diluted 1:500 in 1.5% normal goat serum was used as secondary antibody. After the slides had been rinsed in PBS as before, immunoperoxidase staining was performed. Sections were incubated with the Vectastain Elite ABC reagent (prepared 30–60 minutes before use) for 30 minutes and washed three times with PBS for 5 minutes each time. For peroxidase visualization the DAB Substrate Kit for peroxidase (Vector Laboratories, Burlingame, CA, USA) was used. Color development was stopped by rinsing the sections in water for 5 minutes. Sections were counterstained with hematoxylin for 1 minute and coverslipped with Mowiol (Polysciences, Warrington, PA, USA) or Eukitt (EMS, Fort Washington, PA, USA).

### Microscopy and image processing

Stained sections were analyzed using a widefield microscope (Zeiss Axiovert 200 M) equipped with the AxioCam HRC (Zeiss, Germany) and an Achroplan 10×/0,25 Ph1 or a LD Achroplan 40×/0,60 Korr Ph2 (DICIII) objective. Images were further processed with Axiovision software version 4.5 (Zeiss, Germany).

## Authors' contributions

KE carried out the immunohistochemistry, analyzed the data and helped to draft the manuscript. SL purified the anti-PKD3 polyclonal immune serum. KP was involved in conception of study, data interpretation and manuscript writing. AH designed the study, analyzed and interpreted the data, and drafted the manuscript. All authors read and approved the final manuscript.

## References

[B1] Maier D, Hausser A, Nagel AC, Link G, Kugler SJ, Wech I, Pfizenmaier K, Preiss A (2006). Drosophila protein kinase D is broadly expressed and a fraction localizes to the Golgi compartment. Gene Expr Patterns.

[B2] Maier D, Nagel AC, Gloc H, Hausser A, Kugler SJ, Wech I, Preiss A (2007). Protein kinase D regulates several aspects of development in Drosophila melanogaster. BMC Dev Biol.

[B3] Feng H, Ren M, Rubin CS (2006). Conserved domains subserve novel mechanisms and functions in DKF-1, a C. elegans protein kinase D. J Biol Chem.

[B4] Feng H, Ren M, Wu SL, Hall DH, Rubin CS (2006). Characterization of a novel protein kinase D: C. elegans DKF-1 is activated by translocation-phosphorylation and regulates movement and growth in vivo. J Biol Chem.

[B5] Wang QJ (2006). PKD at the crossroads of DAG and PKC signaling. Trends Pharmacol Sci.

[B6] Hayashi A, Seki N, Hattori A, Kozuma S, Saito T (1999). PKCnu, a new member of the protein kinase C family, composes a fourth subfamily with PKCmu. Biochim Biophys Acta.

[B7] Rey O, Yuan J, Young SH, Rozengurt E (2003). Protein kinase C nu/protein kinase D3 nuclear localization, catalytic activation, and intracellular redistribution in response to G protein-coupled receptor agonists. J Biol Chem.

[B8] Yuan J, Rey O, Rozengurt E (2006). Activation of protein kinase D3 by signaling through Rac and the alpha subunits of the heterotrimeric G proteins G(12) and G(13). Cell Signal.

[B9] Yuan J, Rey O, Rozengurt E (2005). Protein kinase D3 activation and phosphorylation by signaling through G alpha q. Biochem Biophys Res Commun.

[B10] Matthews SA, Dayalu R, Thompson LJ, Scharenberg AM (2003). Regulation of protein kinase Cnu by the B-cell antigen receptor. J Biol Chem.

[B11] Matthews SA, Liu P, Spitaler M, Olson EN, McKinsey TA, Cantrell DA, Scharenberg AM (2006). Essential role for protein kinase D family kinases in the regulation of class II histone deacetylases in B lymphocytes. Mol Cell Biol.

[B12] Hausser A, Storz P, Martens S, Link G, Toker A, Pfizenmaier K (2005). Protein kinase D regulates vesicular transport by phosphorylating and activating phosphatidylinositol-4 kinase IIIbeta at the Golgi complex. Nat Cell Biol.

[B13] Yeaman C, Ayala MI, Wright JR, Bard F, Bossard C, Ang A, Maeda Y, Seufferlein T, Mellman I, Nelson WJ, Malhotra V (2004). Protein kinase D regulates basolateral membrane protein exit from trans-Golgi network. Nat Cell Biol.

[B14] Bossard C, Bresson D, Polishchuk RS, Malhotra V (2007). Dimeric PKD regulates membrane fission to form transport carriers at the TGN. J Cell Biol.

[B15] Fugmann T, Hausser A, Schoffler P, Schmid S, Pfizenmaier K, Olayioye MA (2007). Regulation of secretory transport by protein kinase D-mediated phosphorylation of the ceramide transfer protein. J Cell Biol.

[B16] Sanchez-Ruiloba L, Cabrera-Poch N, Rodriguez-Martinez M, Lopez-Menendez C, Jean-Mairet RM, Higuero AM, Iglesias T (2006). Protein kinase D intracellular localization and activity control kinase D-interacting substrate of 220-kDa traffic through a postsynaptic density-95/discs large/zonula occludens-1-binding motif. J Biol Chem.

[B17] Doppler H, Storz P (2007). A novel tyrosine phosphorylation site in protein kinase D contributes to oxidative stress-mediated activation. J Biol Chem.

[B18] Lu G, Chen J, Espinoza LA, Garfield S, Toshiyuki S, Akiko H, Huppler A, Wang QJ (2007). Protein kinase D 3 is localized in vesicular structures and interacts with vesicle-associated membrane protein 2. Cell Signal.

[B19] Chen J, Lu G, Wang QJ (2005). Protein kinase C-independent effects of protein kinase D3 in glucose transport in L6 myotubes. Mol Pharmacol.

[B20] Oster H, Abraham D, Leitges M (2006). Expression of the protein kinase D (PKD) family during mouse embryogenesis. Gene Expr Patterns.

[B21] Avkiran M, Rowland AJ, Cuello F, Haworth RS (2008). Protein kinase d in the cardiovascular system: emerging roles in health and disease. Circ Res.

[B22] Haworth RS, Goss MW, Rozengurt E, Avkiran M (2000). Expression and activity of protein kinase D/protein kinase C mu in myocardium: evidence for alpha1-adrenergic receptor- and protein kinase C-mediated regulation. J Mol Cell Cardiol.

[B23] Harrison BC, Kim MS, van Rooij E, Plato CF, Papst PJ, Vega RB, McAnally JA, Richardson JA, Bassel-Duby R, Olson EN, McKinsey TA (2006). Regulation of cardiac stress signaling by protein kinase d1. Mol Cell Biol.

[B24] Fielitz J, Kim MS, Shelton JM, Qi X, Hill JA, Richardson JA, Bassel-Duby R, Olson EN (2008). Requirement of protein kinase D1 for pathological cardiac remodeling. Proc Natl Acad Sci U S A.

[B25] Huynh QK, McKinsey TA (2006). Protein kinase D directly phosphorylates histone deacetylase 5 via a random sequential kinetic mechanism. Arch Biochem Biophys.

[B26] Chang S, McKinsey TA, Zhang CL, Richardson JA, Hill JA, Olson EN (2004). Histone deacetylases 5 and 9 govern responsiveness of the heart to a subset of stress signals and play redundant roles in heart development. Mol Cell Biol.

[B27] Zhang CL, McKinsey TA, Chang S, Antos CL, Hill JA, Olson EN (2002). Class II histone deacetylases act as signal-responsive repressors of cardiac hypertrophy. Cell.

